# Degradation Pathway for Eplerenone by Validated Stability Indicating UP-LC Method

**DOI:** 10.5402/2012/251247

**Published:** 2012-10-14

**Authors:** Kondru Sudhakar Babu, Venkataramanna Madireddy, Venkata Somaraju Indukuri

**Affiliations:** ^1^Department of Chemistry, Sri Krishnadevaraya University, Anantapur 515003, Andhra Pradesh 515003, India; ^2^Hetero Labs Ltd., Hetero House, Sanathnagar, 500078 Andhra Pradesh, Hyderabad, India; ^3^InvaGen Pharmaceuticals Inc., Hauppauge 11749, NY, USA

## Abstract

Degradation pathway for eplerenone is established as per ICH recommendations by validated and stability-indicating reverse phase liquid chromatographic method. Eplerenone is subjected to stress conditions of acid, base, oxidation, and thermal and photolysis. Significant degradation is observed in acid and base stress conditions. Four impurities are studied and the major degradant (RRT about 0.31) was identified by LC-MS and spectral analysis. The stress samples are assayed against a qualified reference standard and the mass balance is found close to 99.5%. Efficient chromatographic separation is achieved on a Waters symmetry C18 stationary phase with simple mobile phase combination delivered in gradient mode and quantification is carried at 240 nm at a flow rate of 1.0 mL min^−1^. In the developed LC method the resolution between eplerenone and four potential impurities (imp-1, imp-2, imp-3, and imp-4) is found to be greater than 4.0. Regression analysis shows an *r* value (correlation coefficient) of greater than 0.999 for eplerenone and four potential impurities. This method is capable to detect the impurities of eplerenone at a level of 0.020% with respect to test concentration of 1.0 mg mL^−1^ for a 20 **μ**L injection volume. The developed UPLC method is validated with respect to specificity, linearity and range, accuracy, precision, and robustness for impurities and assay determination.

## 1. Introduction

Eplerenone, pregn-4-ene-7, 21-dicarboxylic acid, 9, 11-epoxy-17-hydroxy-3-oxo, *γ*-lactone, methyl ester (7*α*, 11*α*, 17*α*), is an aldosterone antagonist used as an adjunct in the management of chronic heart failure. It is similar to the diuretic spironolactone, though it may be more specific for the mineralocorticoid receptor. Eplerenone is used alone or in combination with other medications to treat high blood pressure. Eplerenone is in a class of medications called mineralocorticoid receptor antagonists. It works by blocking the action of aldosterone, a natural substance in the body that raises blood pressure. Eplerenone blocks the actions of the hormone aldosterone in the body. Aldosterone is important for the regulation of blood pressure. Eplerenone (other names include SC-66110, CGP-30083, and epoxymexrenone) is the first agent to block the mineralocorticoid receptor with a high degree of selectivity and is under development for human therapeutic use in the 4 treatment of hypertension and heart failure post myocardial infarction [1–5].

A few chromatographic methods have appeared in the literature for the quantification of eplerenone in using SPE-LC-MS/MS for eplerenone and its hydrolyzed metabolite in human urine and development and validation of a liquid chromatography-tandem mass spectrometric assay for eplerenone and its hydrolyzed metabolite in human plasma [6]. One method appeared: “stability-indicating UPLC method for analysis of eplerenone in a bulk drug and pharmaceutical dosage form” [7].

To the best of our knowledge, no stability-indicating LC method for the quantitative estimation of eplerenone in bulk drug substance samples in the presence of degradation products along with its potential impurities has been reported. The purpose of the present research work is to develop a single stability-indicating LC method for the determination of eplerenone and its related impurities and to establish the degradation pathway for eplerenone along with its four potential impurities. The developed LC method is validated with respect to specificity, LOD, LOQ, linearity, precision, accuracy, and robustness. Accordingly the aim of the present study is to establish degradation pathway of eplerenone through stress studies under a variety of ICH-recommended test conditions [8, 9]. 

## 2. Experimental

### 2.1. Chemicals and Reagents

**Figure 1:**
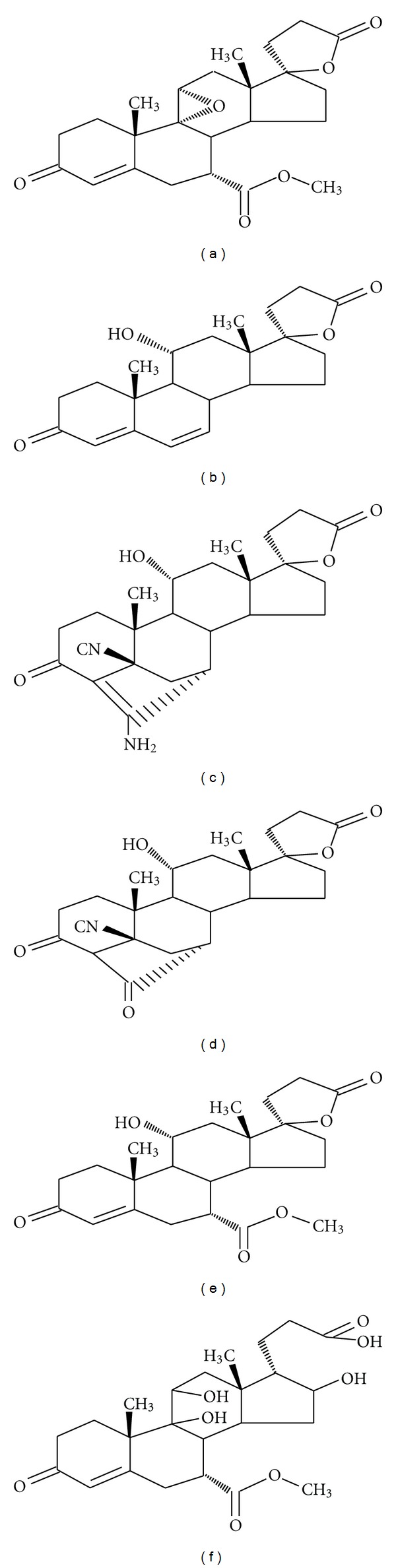
Chemical structures and names of eplerenone and its impurities. (a) Eplerenone: pregn-4-ene-7, 21-dicarboxylic acid, 9, 11-epoxy-17-hydroxy-3-oxo, *γ*-lactone, methyl ester (7*α*, 11*α*, 17*α*) (*molecular weight 414.49*), (b) impurity -1: (11a,17a)-11,17-dihydroxy-3-oxo-pregna-4,6-diene-21-carboxylic acid (molecular weight 356.46), (c) impurity-2 : 5′R(5′alpha),7′beta-20′-amino hexadecahydro-11′beta-hydroxy-10′a,13′alpha-dimethyl-3′,5′-dioxospiro[furan-2(3H),17′alpha(5′H)-[7,4]methano[4H[cyclopenta[a]phenathrene]-5′-carbonitrile (*molecular weight 410*), (d) impurity-3 : 4′S(4′alpha),7′alpha-hexadecahydro-11′alpha-hydroxy-10′beta,13′beta-dimethyl-3′,5,20′-trioxospiro [furan-2(3H),17′beta-[4,7]methanol[17H]cyclopenta[a]phenanthrene]-5′beta(2′H) carbonitrile (*molecular weight 411*), (e) impurity-4: Methyl hydrogen 11 alpha, 17alpha-dihydroxy-3-oxopregn-4-ene-7 alpha, 21- dicarboxylate, gamma-lactone (*molecular weight 416*), and (f) 0.31& 0.86 RRT degradation impurity: methyl hydrogen 9,11 dihydroxy,17-*α*-hydroxy-3-oxopregn 7-*α*-carbonate, 21-*α*-carboxylic acid. (*molecular weight 451*).

Samples of eplerenone and its related impurities are received from Hetero labs limited a research foundation of the firm Hetero Drugs Ltd, Hyderabad, India (Figure 1). All impurities and the eplerenone standard are of > 99% purity and as follows: eplerenone (99.1%), imp-1 (99.5%), imp-2 (95.6%), imp-3 (99.7%), and imp-4 (99.5%). In addition, HPLC grade acetonitrile and methanol are purchased from Merck (Darmstadt, Germany). Analytical reagent grade sodium dihydrogen phosphate monohydrate, phosphoric acid, and acetic acid are purchased from Merck. Highly pure water was prepared with the Millipore Milli-Q Plus water purification system.

### 2.2. Instrumentation

The LC system used for method development, forced degradation studies, and method validation consisted of a Waters 2695 binary pump with an autosampler and a 2996 photodiode array detector (PDA). The output signal is monitored and processed using Empower software on a Pentium computer (Digital equipment Co.). Photostability studies are carried out in a photo stability chamber (Atlas Suntest CPS+). Thermal stability studies are carried out in a dry hot air oven (Cintex precision hot air oven).

### 2.3. Chromatographic Conditions

A Waters symmetry C18 150 mm × 4.6 mm, 5 *μ*m column is used with a mobile phase containing a gradient of solvents A and B. The buffer is composed of 0.1 M ammonium acetate, with its pH adjusted to 3.0 with orthophosphoric acid. Solvents A and B consisted of buffer and acetonitrile in ratios of 800 : 200 (v/v) and 300 : 700 (v/v), respectively. The flow rate of the mobile phase is 1.0 mL/min with a gradient program of 0/10, 10/50, 20/50, 25/10, and 30/10 (time (min)/%B). The column temperature is maintained at 30°C and the detection wavelength is set at 240 nm. The injection volume is 20 *μ*L. The diluent consisted of buffer and acetonitrile in a ratio of 60 : 40 (v/v) (Table 1).

### 2.4. LC-MS Conditions

The LC-MS system (Agilent 2010 EV series liquid chromatography system triple quadrapole mass spectrometer) was used for the identification of unknown compounds formed during forced degradation. A Lichrospher RP 18e 250 × 4.6 mm, 5 *μ*m column was used as the stationary phase. Acetonitrile is used as mobile phase for isocratic mode. Buffer is 0.01 M ammonium acetate and 0.1% triethylamine, pH adjusted to 7.4 using phosphoric acid. The flow rate was 1.0 mL/min. The injection volume is 20 *μ*L. The analysis was performed in positive and negative electrospray ionization modes. The capillary and cone voltages were 4.5 kV and 5 V, respectively. The source and dissolvation temperatures were 250°C and 200°C, respectively, and the dissolvation gas flow is 1.2 mL min^−1^ (Figure 3).

### 2.5. Preparation of Standard Solutions and Sample Solutions

A stock solution of eplerenone (5.0 mg/mL) was prepared by dissolving the appropriate amount of eplerenone solid in the diluent. Working solutions of 0.15% are prepared from the stock solution for the determinations and assays of related substances, respectively. A stock solution of impurities (mixture of imp-1, imp-2, imp-3, and imp-4) at 1.0 mg/mL is also prepared in the diluent. The drug substance powder equivalent to 100 mg of sample is transferred into a 100-mL volumetric flask, and 70 mL of diluent is added. The flask is attached to a rotary shaker and shaken for 10 min to disperse the powder completely. The mixture is sonicated for 10 min and then diluted to the appropriate volume with diluent to make a solution containing 1.0 mg/mL. The solution is then filtered through a 0.45 *μ* Nylon 66 membrane filter.

### 2.6. Stress Studies/Specificity

Specificity is the ability of the method to measure the analyte response in the presence of its potential impurities. [10, 11] The specificity of the developed RPLC method for eplerenone is determined in the presence of its related impurities (namely, imp-1, imp-2, imp-3, and imp-4) and degradation products. Forced degradation studies are also performed on eplerenone to provide an indication of the stability-indicating property and specificity of the proposed method. [12–16].

The stress conditions employed for the degradation study included light (carried out as per ICH Q1B), heat (100°C), acid hydrolysis (1 M HCl), base hydrolysis (0.5 M NaOH) and oxidation (15% H_2_O_2_). For heat and light studies, the samples were exposed for 168 hrs, whereas the samples are treated for 2 h for acid, base hydrolysis, and for oxidation. The peak purity of the eplerenone stressed samples is also checked by using a Waters 2996 photo diode array detector (PDA). The purity angle is within the purity threshold limit in all of the stressed samples, demonstrating the homogeneity of the analyte peak. The contents of impurities were calculated for the stress samples against a qualified reference standard. The mass balance (% assay + % of impurities + % of degradation products) is calculated for all of the samples (Table 2).

### 2.7. Method Validation

The proposed method was validateds per ICH guidelines [10].

#### 2.7.1. Precision

The precision of the related substance method is investigated by injecting six individual preparations of (5 *μ*g/mL) eplerenone spiked with 0.05% each of imp-1, imp-2, imp-3, and imp-4. The %RSD of the areas of imp-1, imp-2, imp-3, and imp-4 is calculated.

The intermediate precision of the method is evaluated using a different analyst and instrument located within the same laboratory.

 The precision of the assay method is evaluated by carrying out six independent assays of a test sample of eplerenone against a qualified reference standard. The %RSD of six obtained assay values is calculated.

#### 2.7.2. Limit of Detection (LOD) and Limit of Quantification (LOQ)

The LOD and LOQ for imp-1, imp-2, imp-3, and imp-4 are estimated at a signal-to-noise ratio of 3 : 1 and 10 : 1, respectively, by injecting a series of dilute solutions with known concentrations. The precision study is also carried out at the LOQ level by injecting six individual preparations of imp-1, imp-2, imp-3 and imp-4 and calculated the %RSD of the areas.

#### 2.7.3. Linearity and Range

 Linearity test solutions for the assay method are prepared from a stock solution at six concentration levels from 50 to 150% of the analyte concentration (50, 75, 100, 125, and 150). The peak area versus concentration data is analyzed with least-squares linear regression. Linearity test solutions for the related substance method are prepared by diluting the impurity stock solution (2.5) to the required concentrations. The solutions are prepared at eight concentration levels from the LOQ to 150% (LOQ, 0.075%, 0.12%, 0.15%, 0.18%, and 0.225%). The slope and y-intercept of the calibration curve are reported (Table 3) (Figure 4). The peak area versus concentration data is analyzed with least-squares linear regression. Linearity test solutions for the related substance method were prepared by diluting the impurity stock solution (2.5) to the required concentrations. The solutions are prepared at six concentration levels from the LOQ to 150% (LOQ, 0.075%, 0.12%, 0.15%, 0.18%, and 0.225%). The slope and y-intercept of the calibration curve are reported (Table 3).

#### 2.7.4. Accuracy

Accuracy of the assay method is evaluated in triplicate at three concentration levels 50, 100, and 150, and the percentage recoveries are also calculated. Eplerenone did not show the presence of imp-2 imp-4, but contained 0.03% of imp-1, 0.06% of imp-3. Standard addition and recovery experiments are conducted to determine the accuracy of the related substance method for the quantification of all four impurities (imp-1, imp-2, imp-3, and imp-4) in the drug substance. The study was carried out in triplicate at 0.075%, 0.15%, and 0.225% of the analyte concentration (5.0 *μ*g/mL). The percentage of recoveries for imp-1, imp-2, imp-3, and imp-4 is calculated (Table 4).

#### 2.7.5. Robustness

 To determine the robustness of the developed method, the experimental conditions are altered and the resolution between eplerenone and imp-1, imp-2, imp-3, and imp-4 is evaluated. The flow rate of the mobile phase is 1.0 mL/min. To study the effect of the flow rate on the resolution, the flow rate is changed by 0.2 units (to 0.8 and 1.2 mL/min). The effect of pH on the resolution of the impurities is studied by varying the pH by ±0.2 units (buffer pH of 4.3 and 4.7). The effect of the column temperature on the resolution is studied at 25°C and 35°C instead of 30°C. In all these varied conditions, the components of the mobile phase remained constant, as outlined in Section 2.3.

### 2.8. Solution Stability and Mobile Phase Stability

**Figure 2:**
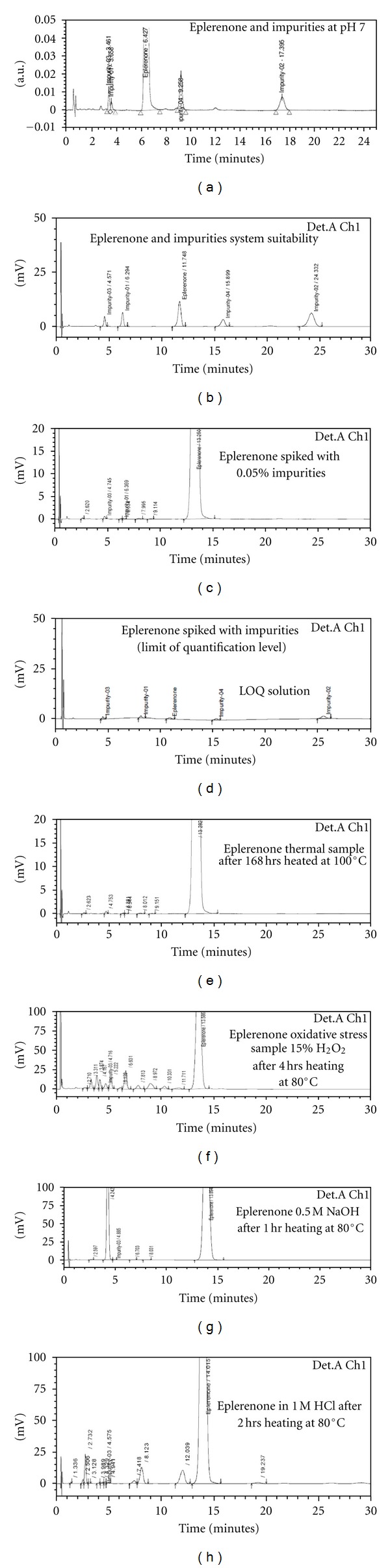
Typical chromatogram from the method development trials optimized conditions and stressed eplerenone samples.

**Figure 3:**
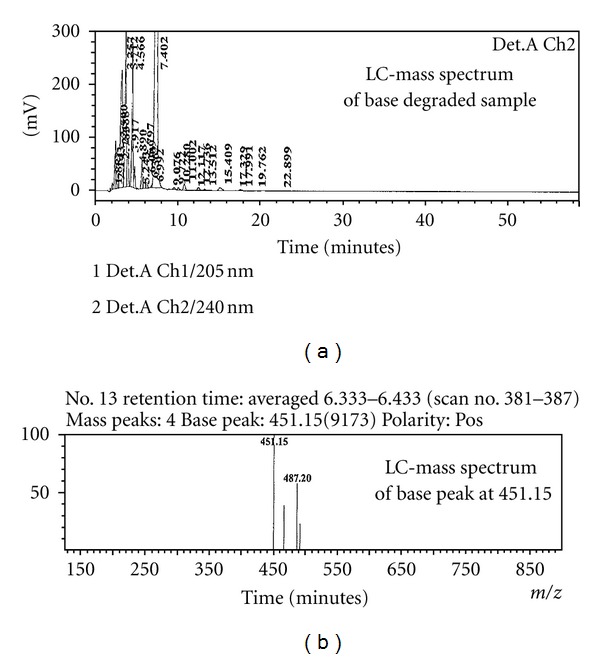
LC-mass chromatograms.

**Figure 4:**
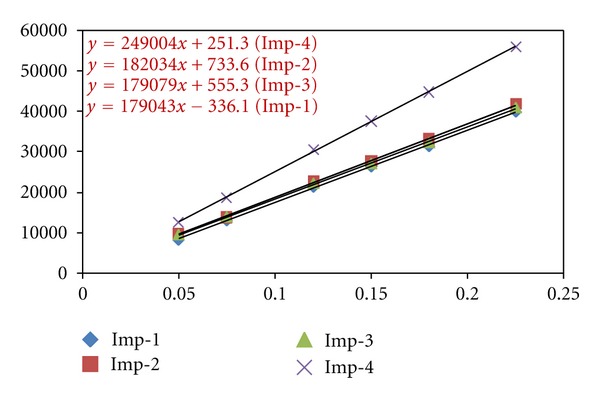
Linearity chart for eplerenone impurities.

 The solution stability of eplerenone in the assay method is carried out by leaving both the sample and reference standard solutions in tightly capped volumetric flasks at room temperature for 48 h. The same sample solutions are assayed for in 6 h intervals over the study period. The mobile phase stability was also examined by assaying the freshly prepared sample solutions against freshly prepared reference standard solutions for 6 h intervals up to 48 h. The prepared mobile phase remained constant during the study period (Figure 2). The %RSD of the eplerenone assay is calculated for the mobile phase and solution stability experiments. The solution stability of eplerenone and its impurities in the related substance method is carried out by leaving a spiked sample solution in a tightly capped volumetric flask at room temperature for 48 h. The content of imp-1, imp-2, imp-3, and imp-4 is determined at 6 h intervals up to the study period. The mobile phase stability is also investigated for 48 h by injecting the freshly prepared sample solutions for every 6 h interval. The content of imp-1, imp-2, imp-3, and imp-4 is determined in the test solutions. The prepared mobile phase remained constant during the study period.

## 3. Results and Discussion

### 3.1. Method Development and Optimization

 The main objective of the chromatographic method is to separate imp-1, imp-2, imp-3, imp-4, eplerenone, and the generated degradation products from the analyte peak during stress studies. Impurities and degradation products are coeluted by using different stationary phases, such as C8, cyano, and phenyl, with various mobile phases with buffers, such as phosphate, sulphate and acetate with different pH values (2–5), and organic modifiers, including acetonitrile and methanol, in the mobile phase. 0.1 M ammonium acetate buffer with a pH value of 2.4 and methanol (50 : 50, v/v) at a flow rate of 1.2 mL/min is chosen for the initial trail with a 100 mm × 4.6 mm ID column and 1.8 *μ*m particle size C18 stationary phase. When an impurity-spiked solution is injected, the resolution between the impurities and analyte is poor. Imp-1 and imp-3 are almost co-eluted with the analyte. To improve the resolution between the impurities and analyte, acetonitrile is replaced with methanol in the mobile phase and flow rate was slightly changed and injected into the impurity-spiked solution. The resolution between the impurities and analyte was slightly improved but one of the impurities is not eluted even after 40 min of run time. To optimize the resolution between the impurities and the retention time of the impurities, trails are carried out with different mobile phase ratios using buffer and acetonitrile (buffer: acetonitrile: 90 : 10, 80 : 20, 70 : 30 v/v). Isocratic trials are not successful in achieving a favorable resolution between impurity and analyte peaks and the elution of the process impurities. Therefore, a gradient method is selected using buffer and acetonitrile in a ratio of 80 : 20, 30 : 70(v/v) as mobile phase A and water and acetonitrile in a ratio of 30 : 70 (v/v) as mobile phase B. Different gradient programs were investigated and satisfactory results are obtained when a gradient program of 0/10, 10/50, 20/50, 25/10, and 30/10 (time (min)/%B) is used.

### 3.2. Results of Forced Degradation Studies

Degradation was not observed in eplerenone stressed samples subjected to light and heat. Significant degradation of the drug substance and product is detected under acid and base hydrolysis, leading to the formation of one major unknown degradation product at 0.31 RRT (Figure 2). Peak purity test results derived from the PDA detector confirmed that the eplerenone peak and the degraded peaks are homogeneous and pure in all of the analyzed stress samples. Degradation studies are carried out for the stress samples (at 100 *μ*g/mL) against a qualified reference standard of eplerenone (Figure 2).

The mass balance of the stressed samples was close to 99.5%. The assay of eplerenone is unaffected by the presence of imp-1, imp-2, imp-3, imp-4, and its degradation products, confirming the stability-indicating power of the developed method.

### 3.3. Identification of Major Degradation Product (Rrt 0.31) Formed in Base Hydrolysis (*Stress Conditions*)

An LCMS study was carried to determine the *m/z *value of the major degradation product formed under acid and base hydrolysis using an Agilent 2010 EV series liquid chromatography system coupled with triple quadrapole mass spectrometer. Acetonitrile is used as mobile phase for isocratic mode. Buffer is 0.01 M ammonium acetate and 0.1% triethylamine, pH adjusted to 7.4 using phosphoric acid. The *m/z* value obtained for the degradation product resolving at 0.31 and 0.86 RRT in ESI-positive mode was 451(M+1). The impurity was isolated using preparative LC-MS. Based on the mass number the identified degradant is methyl hydrogen 9, 11 dihydroxy, 17-*α*-hydroxy-3-oxopregn 7-*α*-carbonate, 21-*α*-carboxylic acid with molecular weight of 451.10 (Figure 1).

### 3.4. Results of Method Validation

#### 3.4.1. Precision

The %RSD of eplerenone during the method precision study is within 3.0% and the %RSD values of the area of imp-1, imp-2, imp-3, and imp-4 in the related substance method precision study are within 10.0%. The %RSD of the results obtained in the intermediate precision study was within 3.0% and the %RSD of the areas of imp-1, imp-2, imp-3, and imp-4 were well within 5%, revealing the high precision of the method (Table 3).

#### 3.4.2. Limit of Detection and Limit of Quantification

The limits of detection and quantification of eplerenone, imp-1, imp-2, imp-3, and imp-4 for a 20-*μ*l injection volume are given in Table 3. The precision at the LOQ concentration for imp-1, imp-2, imp-3, and imp-4, is below 10.0%.

#### 3.4.3. Linearity and Range

 The linear calibration plot for the method is obtained over the tested calibration range (50%—150% level) and the obtained correlation coefficient is greater than 0.999. The results revealed an excellent correlation between the peak area and analyte concentration. The linear calibration plot for the related substance method is determined over the calibration range (LOQ to 0.225% with respect to analyte concentration) for imp-1, imp-2, imp-3, and imp-4, a correlation coefficient of greater than 0.999 is obtained. The linearity was checked for the related substance method over the same concentration range for three consecutive days. The %RSD values of the slope and y-intercept of the calibration curves are within 15.0%. These results showed an excellent correlation between the peak areas and concentrations of imp-1, imp-2, imp-3, and imp-4 (Table 3). Residuals are within ±10% scattered with respect to 100% concentration response. Sensitivities are scattered within ±10% with respect to 100% concentration sensitivity.

#### 3.4.4. Accuracy

The percentage recovery of eplerenone impurities in the drug substances, that is, imp-1, imp-2, imp-3, and imp-4, ranged from 94.99 to 105.45, from 96.29 to 101.33, 96.45 to 105.26, and from 95.77 to 105.88, respectively. The UPLC chromatograms of spiked samples at the 0.15% level of all four impurities in the eplerenone drug substance sample are shown in Figure 2.

#### 3.4.5. Robustness

In all of the deliberately varied chromatographic conditions carried out as described in Section 2.7.5 (flow rate, pH, and column temperature), the resolution between the closely eluting impurities, namely, imp-3 and imp4, is greater than 5.0, illustrating the robustness of the method. The variability of eplerenone and the impurities area response is within ±2% and within ±3%, respectively.

#### 3.4.6. Solution Stability and Mobile Phase Stability

The %RSD of assaying eplerenone during the solution stability and mobile phase stability experiments is within 1%. No significant changes are observed in the content of imp-1, imp-2, imp-3, and imp-4 during the solution stability and mobile phase stability experiments when performed using the related substances method. The results of the solution and mobile phase stability experiments confirm that the sample solutions and mobile phase used during the related substance determinations are stable up to 48 h. Mobile phase is proved to be stable up to five days.

## 4. Conclusion

 The degradation pathway of eplerenone is established as per ICH recommendations. The gradient UPLC method developed and used for stress studies also fit for quantitative, related substance and assay determination of eplerenone. The behavior of eplerenone under various stress conditions is studied, and the hydrolysis (base) degradant is identified as “methyl hydrogen 9, 11 dihydroxy, 17-*α*-hydroxy-3-oxopregn 7-*α*-carbonate, 21-*α*-carboxylic acid” by LCMS and presented. All the degradation products and process impurities are well separated from the drug substance which demonstrates the stability-indicating power of the RP-LC analytical method. The method is validated as per ICH guidelines. 

## Figures and Tables

**Table 1:** System suitability report.

Compound	USP resolution (*R*_*S*_)	USP tailing factor	No. of theoretical plates (USP tangent method)
Impurity-3	—	1.2	18945
Impurity-1	5.8	1.2	20542
Eplerenone	12.8	1.4	10376
Impurity-4	6.9	1.2	8209
Impurity-2	9.8	1.4	15035

**Table 2:** Summary of forced degradation results.

Stress condition	% Total impurities	Study time	% Assay of active substance	Mass balance (% assay + % impurities + % degradation products)	Remarks
Acid hydrolysis (1 M HCI)	8.4%	2 h	91.4	99.8	Prominent degradation observed
Base hydrolysis (0.5 M NaOH)	19.8%	1 h	79.7	99.5	One major degradation product was formed RRT about 0.31 and identified as “Methyl hydrogen 9, 11 dihydroxy, 17-*α*-hydroxy-3-oxopregn 7-*α*-carbonate, 21-*α*-carboxylic acid”
Oxidation (15% H_2_O_2_)	19.2%	4 h	78.5	97.7	Prominent Degradation observed
Thermal (100^°^C)	0.3%	7 days	99.3	99.6	No degradation products formed
Light (photolytic degradation)	0.4%	1200 KLUX/Hr	99.1	99.5	No degradation products formed

**Table 3:** Regression and precision data.

Parameter	Eplerenone	Imp-1	Imp-2	Imp-3	Imp-4
LOD (%)	0.007	0.017	0.016	0.020	0.018
LOQ (%)	0.02	0.05	0.05	0.05	0.05
Slope (m)	203310	179043	182034	179079	249004
Intercept (C)	472.93	−336.05	733.64	555.31	251.25
Correlation coefficient	0.99903	0.99977	0.99975	0.99976	0.99990
Precision (%RSD)^a^	3.0	4.0	2.8	1.9	2.3

Linearity range was LOQ-150% with respect to 1.0 mg/mL eplerenone for impurities; linearity range was 50–150% of eplerenone. ^a^Six determinations using LOQ solutions for impurities and eplerenone.

**Table 4:** Batch analysis data.

Lot no.	Imp-1	Imp-2	Imp-3	Imp-4	Max. single unknown Imp.	Total impurities	Assay by HPLC
EPL001	0.03	ND	0.06	ND	0.06	0.17	99.6
EPL002	0.05	ND	0.08	ND	0.05	0.18	99.9
EPL003	0.03	NDN	0.07	ND	0.06	0.19	100.1
